# Survey of Medical Trainees Indicates a Need for Increased Access to Obstetrics and Gynecology Care

**DOI:** 10.7759/cureus.67242

**Published:** 2024-08-19

**Authors:** Rachel J Scheub, Monali S Ardeshna, Helena J Randle, Alicia Wiczulis

**Affiliations:** 1 General Surgery, Yale New Haven Hospital, New Haven, USA; 2 Obstetrics and Gynecology, Albany Medical Center, Albany, USA; 3 Obstetrics and Gynecology, University of Maryland Medical Center, Baltimore, USA

**Keywords:** female physicians, access to obgyn care, medical trainee health, infertility in physicians, women’s health

## Abstract

Introduction: Given the higher rates of infertility and complicated pregnancies among female physicians, we identified a need to assess access to obstetrics and gynecology (OBGYN) care for medical trainees. We hypothesized that medical students and residents are not up-to-date on routine OBGYN care.

Methods: We administered an optional, anonymous survey to all medical students and residents at Albany Medical College (Albany, NY, USA) who self-identified as having a uterus to assess their access to gynecologic care in November 2022. Data collected included demographic information, care-seeking practices, reproductive health screening history, contraception use, and menstrual cycle irregularities.

Results: A total of 184 trainees responded to the survey; 71% were medical students and 29% were residents. Around 11% of respondents had never seen an OBGYN provider. About 45% of respondents had not seen a provider in the last year, 20% had not seen a provider in the last three years, and 37% had not seen a provider since beginning their training. Of the trainees, 26% were not up to date on recommended cervical cancer screening; 35% indicated they had irregular menses; and 50% had not received sexually transmitted infection (STI) testing in the last year. Older age was associated with a lower rate of STI testing. Age and trainee type were both associated with having ever seen an OBGYN provider; both older participants and residents were more likely than younger participants and medical students to have answered 'yes.' Race was also associated with having ever seen an OBGYN provider.

Conclusions: Trainees accessed OBGYN care at lower-than-expected rates. There is an opportunity to improve access to OBGYN care for these trainees, which should be recommended to improve reproductive health in this group.

## Introduction

Medical trainees have challenging careers that can negatively impact their health, with contributing factors such as burnout, long work hours, and fatigue. Lack of routine healthcare may further exacerbate this. A study at a U.S. academic medical center in Oregon stated that only 39% of their residents reported having routine primary care visits, compared to 63% of a group of demographically similar peers [1]. Given the previous data regarding trainees’ utilization of primary care, we predicted that many medical students and residents are not up to date on routine gynecologic care. No studies analyzing medical trainees’ access to and routine compliance with gynecologic care specifically were identified.

A military medical center found that, compared to their male counterparts, female residents were more likely to miss routine health appointments [2]. Previous studies also demonstrated that many residents would prefer to seek gynecologic or sexually transmitted infection (STI)-related care outside of their institution [3]. However, little is known about the factors affecting access to and regularity of gynecologic care. Routine gynecologic care is important, considering most medical students and residents are of reproductive age. Several studies have found higher rates of infertility and complicated pregnancies in female physicians compared with the general female population [4,5]. Improved access to routine screenings and gynecologic care during medical school and residency may allow for earlier diagnosis and treatment of conditions that affect fertility.

In a study of internal medicine residency programs within the United States, 30% of residents reported holding back information about their sexual history from a primary care provider due to privacy and confidentiality concerns [6]. Similar concerns and practices may exist for medical students and residents in terms of their gynecologic care. Previous studies have also demonstrated that a large proportion of residents seek informal medical care from a colleague [7,8]; this trend may be occurring with OBGYN care as well, due to time constraints and confidentiality concerns.

At the study site, all trainees are required to have adequate health insurance, and medical students are offered a comprehensive plan. Additionally, Albany Medical College (Albany, NY, USA) has a health reimbursement program for medical students that reimburses certain out-of-pocket healthcare expenses not covered by insurance. Despite these efforts to reduce financial barriers to accessing care, low rates of gynecologic care within the trainee population were still anticipated.

This study aims to characterize medical trainees’ care-seeking practices for gynecologic care. An anonymous survey was sent to medical students and residents at Albany Medical College to assess their compliance with routine gynecologic care, including STI and cervical cancer screening, evaluation of menstrual irregularities, and access to contraception (if needed or desired). The study also aimed to evaluate if medical students and residents were getting access to medications and contraceptive pills without seeing a provider in person or via telehealth.

## Materials and methods

Survey and design

An optional and anonymous survey was developed to assess access to gynecologic care for trainees. The survey was administered in November 2022 to all medical students and residents at Albany Medical College who self-identified as having a uterus. This was gender-inclusive and excluded trainees who may have had a hysterectomy and would therefore be seeking more individualized care. Given the intimate nature of the questions, no survey questions were required for respondents. For the purpose of this survey, an OBGYN provider was defined as “a physician or advanced practice provider who performs pelvic exams, pap smears, contraception counseling, etc. These services are typically provided in an OBGYN practice but may be provided by some primary care providers.” This study was approved by the institutional review board of Albany Medical College (approval no. 6536). Data collected included demographic information, care-seeking practices, reproductive health screening history, contraception use, and menstrual cycle irregularities. See Appendix A for the questionnaire shared with participants. Within the same survey, potential barriers to care were assessed, and this data will be reported separately.

The survey asked trainees if they had ever seen an OBGYN provider and if they had seen a provider in the last 12 months, three years, or since beginning training. Based on current guidelines, respondents were stratified by age and asked if they had received a pap smear in the last three or five years. Trainees were asked about sexual activity, STI testing, contraception and condom use, STI risk factors, and menstrual irregularities.

Statistics

Associations between demographic data and trainee answers were explored using chi-square analysis (Fisher’s exact test was used for expected values less than five). Participants who did not answer both questions or who selected 'prefer not to answer' for one or both questions being compared were excluded from the comparison. For analyses by race, 'multiracial/other' was combined and was defined as anyone who selected either 'other' or selected two or more answers (e.g., white and Asian were both selected). Sexuality was grouped for comparison into straight/heterosexual and lesbian, gay, bisexual, transgender, and queer (LGBTQ+), which consisted of anyone who selected bisexual, gay or lesbian, 'I don’t know,' or pansexual. For analyses by age, under 21 and 21 to 24 were grouped together as one age group, as well as 33 to 39 and 40+. 

## Results

Trainee characteristics

A total of 184 trainees responded to the survey (Table 1); 131 (71%) were medical students and 53 (29%) were residents. All respondents self-identified as having a uterus. The medical student cohort consisted of 38 (32%) medical school year-1 (MS1s), 30 (25%) MS2s, 30 (25%) MS3s, and 21 (18%) MS4s. The resident cohort consisted of 11 (21%) postgraduate year-1 students (PGY1s), 13 (25%) PGY2s, 10 (19%) PGY3s, 15 (29%) PGY4s, and 3 (6%) PGY5+. Of the survey respondents, 138 (81%) were <29 years old, and 33 (19%) were 30 years old or greater; 34 (29%)students and 47 (92%) residents were on an insurance plan offered by the school. Of the respondents, 103 (59%) identified as white, 10 (6%) identified as Black or African American, two (1%) of the respondents identified as American Indian or Alaska Native, 44 (25%) identified as Asian, 14 (8%) identified as other, and 16 (10%) identified as Hispanic or Latino. Among the participants, 165 (98%) identified as female, four (2%) identified as either male, non-binary, genderqueer, or genderfluid, and 132 (79%) identified as straight or heterosexual. The sample included students and residents from multiple subspecialties, with the greatest representation of students and residents interested in OBGYN.

**Table 1:** Demographic data of participants MS: Medical school year, PGY: Postgraduate year, PM&R: Physical medicine and rehabilitation, OBGYN: Obstetrics and gynecology, FM: Family medicine, IM: Internal medicine, IR: Interventional radiology, Rad/Onc: Radiation oncologist, Med-Peds: Internal medicine-pediatrics

Variable		Total number (n)	Percentage
Type of trainee			
	Medical students	131	71%
	Resident	53	29%
	Total	184	
Year of medical school			
	MS1	38	32%
	MS2	30	25%
	MS3	30	25%
	MS4	21	18%
	Total	119	
Year of residency			
	PGY1	11	21%
	PGY2	13	25%
	PGY3	10	19%
	PGY4	15	29%
	PGY5	2	4%
	PGY6+	1	2%
	Total	52	
Age			
	Under 21	1	0.50%
	21-24	48	28%
	25-29	89	52%
	30-32	23	13%
	33-39	9	5%
	40+	1	0.50%
	Total	171	
On student AMC health insurance?			
	Yes	34	29%
	No	84	71%
	Unsure	1	1%
	Total	119	
On resident AMC health insurance?			
	Yes	47	92%
	No	4	8%
	Total	51	
Race			
	White	103	59%
	Black or African American	10	6%
	American Indian or Alaska Native	2	1%
	Asian	44	25%
	Other	14	8%
	Prefer not to answer	5	3%
	Total	178	
Ethnicity			
	Hispanic or Latino	16	10%
	Non-Hispanic or Latino	147	88%
	Prefer not to answer	4	2%
	Total	167	
Gender identity			
	Male	2	1%
	Female	165	98%
	Non-binary	1	0.50%
	Genderqueer or genderfluid	1	0.50%
	Total	169	
Sexual orientation			
	Bisexual	20	12%
	Gay or lesbian	5	3%
	Straight/heterosexual	132	79%
	Pansexual	4	2%
	I don't know	3	2%
	Prefer not to answer	3	2%
	Total	167	
Subspecialty interest in students (up to 3)			
	Anesthesiology	13	6%
	Dermatology	7	3%
	Emergency medicine	19	9%
	General surgery and/or surgical subspecialty	28	13%
	Neurology and/or PM&R	14	6%
	OBGYN	40	19%
	Ophthalmology	5	2%
	Primary Care (FM, Peds, and/or IM)	64	30%
	Psychiatry	13	6%
	Radiology (Diagnostic, IR, Rad/Onc)	4	2%
	Other	7	3%
	Unsure	2	1%
	Total	216	
Department (resident)			
	Anesthesiology	1	2%
	Emergency medicine	2	4%
	Family medicine	2	4%
	General surgery	3	6%
	IM/ Med-Peds	6	12%
	Neurology	3	6%
	OBGYN	10	20%
	Ophthalmology	2	4%
	Pathology	1	2%
	Pediatrics	5	1%
	Psychiatry	5	1%
	PM&R	1	2%
	Radiology (Diagnostic, IR)	2	4%
	Surgical Subspecialty	6	12%
	Total	49	

Access to care

Of the respondents, 19 (11%) indicated that they had never seen an OBGYN provider in their lifetime, 76 (45%) had not seen a provider in the last year, 34 (20%) had not seen a provider in the last three years, and 55 (37%) had not seen a provider since beginning their training. Around 49 (53%) respondents switched to a new OBGYN provider when initiating their training. Of the trainees, 40 (26%) were not up-to-date on recommended cervical cancer screening. About 55 (35%) trainees indicated they had irregular menses, and of these, 18 (33%) indicated they did not have a condition or medication that would cause them to have irregular menses. Also, 43 (28%) trainees had ordered medications online; of these, 20 (47%) had ordered contraception online.

Testing for STIs

Around 31 (20%) trainees reported that they had never been tested for STIs in their lifetime, and of this subset, 22 (71%) reported that they had been sexually active in the past. Of the trainees, 64 (50%) had not received STI testing in the last year, and 11 (33%) of them aged <25 in our sample had not had STI testing in the last year; 13 (20%) of those who had not received STI testing in the last year had sex with a new partner since last being STI-tested. Of the participants, 128 (85%) did not consider themselves to be at risk of STIs (indicated by a selection of 'definitely not' or 'probably not' when asked if they considered themselves to be at risk). Of those who did not consider themselves at risk, 100 (78%) indicated they were in a monogamous relationship; however, 48 (48%) of those in monogamous relationships were either unsure or indicated that their partner had not been tested since previous partners; 77 (77%) of those in a monogamous relationship did, however, indicate that they had been tested themselves since previous partners. When asked on a 5-point Likert scale ranging from 'always' to 'never,' 64 (43%) participants indicated they never used condoms, and 28 (19%)indicated they always did.

Associations between demographic characteristics and OBGYN care-seeking behaviors

Age and trainee type were both associated with having ever seen an OBGYN provider; both older participants and residents were more likely than younger participants and medical students to have answered 'yes' (p = 0.0161, 0.0476, respectively) (Table 2). Race was also a modifier of having ever seen an OBGYN provider (p = 0.0003); a greater number of trainees who selected Asian had never seen an OBGYN provider. In terms of STI testing, age was associated with having received STI testing in the last year; older age was associated with a decreased rate of STI testing (Table 3). Otherwise, age, sexuality, race, ethnicity, or trainee type were not modifiers of the results reported above.

**Table 2:** Association between trainee type, age, race, ethnicity, and sexuality and having ever seen an OBGYN provider *Statistically significant chi-square/Fisher's exact (p < 0.05) OBGYN: Obstetrics and gynecology, LGBTQ: Lesbian, gay, bisexual, transgender, queer

Have you EVER seen an OBYN provider?		No	Yes	Total
Trainee type* (p = 0.0476)				
	Medical student	17	101	118
	Resident	2	49	51
	Total	19	150	169
Age* (p = 0.0161)				
	≤ 24	9	39	48
	25-29	8	81	89
	30-32	0	22	22
	≥ 33	2	8	10
	Total	19	150	169
Race* (p = 0.0003)				
	White	4	90	94
	Asian	12	27	39
	Black/African American	1	8	9
	Other/Multi	1	20	21
	Total	18	145	163
Ethnicity (p = 0.6832)				
	Hispanic or Latino	2	14	16
	Non-Hispanic or Latino	16	129	145
	Prefer not to answer	1	3	4
	Total:	19	146	165
Sexuality (p = 0.3343)				
	LGBTQ+	2	30	35
	Straight/heterosexual	15	117	132
	Total	17	147	164

**Table 3:** Association between trainee type, age, race, ethnicity, and sexuality and having received STI testing in the last year *Statistically significant chi-square/Fisher's exact (p < 0.05) STI: Sexually transmitted infection, LGBTQ: Lesbian, gay, bisexual, transgender, queer

Have you received STI testing in the last year?		No	Yes	Total
Trainee type (p = 0.0882)				
	Medical student	39	48	87
	Resident	25	16	41
	Total	64	64	128
Age* (p = 0.0308)				
	≤ 24	11	22	33
	25-29	37	34	71
	30-32	13	4	17
	≥ 33	3	4	7
	Total	64	64	128
Race (p = 0.4357)				
	White	40	39	79
	Asian	13	9	22
	Black/African American	4	4	8
	Other/Multi	6	12	18
	Total	63	64	127
Ethnicity (p = 0.8037)				
	Hispanic or Latino	7	8	15
	Non-Hispanic or Latino	55	53	108
	Prefer not to answer	1	2	3
	Total:	63	63	126
Sexuality (p = 0.9276)				
	LGBTQ+	13	13	26
	Straight/heterosexual	51	49	100
	Total:	64	62	126

## Discussion

This study sought to evaluate medical trainees’ compliance with gynecologic care. According to this study, many trainees were not up-to-date on recommended gynecologic care. The American College of Obstetricians and Gynecologists recommends visiting the OBGYN once a year for a well-woman visit [9]. However, 45% of respondents had not seen an OBGYN in the past year. This proportion is similar to that of U.S. women who saw an OBGYN within the last 12 months, which ranged from 38.4% to 45% between 2000 and 2015 [10].

Current cervical cancer screening guidelines recommend screening with cytology every three years between the ages of 21 and 29 years and co-testing with cytology and screening for high-risk human papillomavirus every five years between the ages of 30 and 65 years [11]. Of our study respondents, only 75% were up to date with these recommendations. By comparison, 88% of women aged 18 years and over with a bachelor’s degree have had a Pap test within the last three years [12]. The 13% difference between trainees and non-trainees suggests additional barriers are impeding trainees from adhering to recommended cervical cancer screening guidelines.

Additionally, half of the respondents had not received STI testing in the past year. Most respondents were above the age of 25, thus testing for STIs such as chlamydia and gonorrhea is only recommended if the person is sexually active and at increased risk (i.e., a new partner, more than one sex partner, a sex partner with concurrent partners, or a sex partner who has an STI). However, based on current recommendations and if sexually active, 33% of respondents under the age of 25 who have not been tested in the last year are not up to date with the current recommendation for yearly screening for certain STIs [13].

A large portion of trainees sought advice about reproductive or sexual health from a colleague. This suggests that they may not be obtaining comprehensive, confidential advice and care that might otherwise be provided during scheduled appointments with physicians. Prior studies found that medical students face barriers to accessing healthcare due to factors such as time constraints and privacy concerns, and that gynecologic concerns were considered stigmatizing by many students [14]. Trainees are in a unique position when seeking medical care while also training to provide medical care themselves. By seeking medical care at their training institution, they may feel as though their professional and personal matters are being intertwined. It is probable that the stigma around gynecologic care has perpetuated trainees’ worries about people involved in their training also being involved in their gynecologic care.

An estimated 1 in 4 female physicians suffers from infertility [4], and physicians are twice as likely as women of reproductive age in the general population to experience infertility [15]. It is important that trainees who may want to have children in the future receive care. Studies have found that physicians initiate childbearing later than non-physicians, in part due to medical training coinciding with prime reproductive years [15]. Additionally, this study found that 35% of trainees had irregular menses, of which 32% did not have a condition or medication that would cause them to have irregular menses. Trainees must receive a work-up for this presentation to reduce risks to their future fertility and identify and treat any associated comorbidities.

Even at an institution with student health services for primary care and a health reimbursement program, there continues to be a delay in access to care. Despite efforts to reduce financial barriers to healthcare, trainees still access care at lower rates than a comparable population. Studies have found that medical students and residents alike face time constraints and privacy concerns when seeking medical care [6,7,14]. Receiving care at their institution should, in theory, decrease the time burden of accessing gynecologic care through means such as transit time and searching for a provider. However, if trainees continue to have time burdens and privacy concerns when receiving care at their institutions, trainees may forgo receiving care at all so that they do not risk peers accessing their health information or working in the future with individuals involved with their gynecologic care.

There is a need for increased education surrounding how to access care and the risks of not receiving care. Similar patterns are likely present at other medical institutions and in parallel work fields. Studies show that the personal health behaviors of physicians have a direct impact on their clinical practice [16]. Thus, as the future generation of physicians, trainees must maintain their health and set proper examples for other trainees and patients as they carry on throughout their training and practice. If barriers continue to persist, a culture of avoiding or delaying gynecologic care will be perpetuated. To increase access to gynecologic care and protect trainees’ reproductive health, the unique barriers that trainees face need to be identified and addressed. We recommend increasing awareness on how to access gynecologic care throughout training, the significance of care, and the ramifications of not receiving it, with consideration of trainee confidentiality and scheduling difficulties.

The limitations of this study need to be considered when interpreting and generalizing the results. The data for this study was collected via a self-reported questionnaire, and thus participants chose whether to fill out the survey and be a part of the sample. This has the potential to introduce biases if participants have specific motivations for filling out the form. Additionally, although responses were anonymous, it is possible that participants were less willing to disclose all pertinent personal information knowing that it was colleagues conducting the study. The sample size for this study was small and limited to one academic medical institution in the Northeast. It is possible that the results are, at least in part, institutionally and geographically dependent. The participants are also in multiple points of training; whether trainees were in pre-clinical years or clinical years may have impacted the results of this study and should be considered further. Due to these limitations, generalizing the results of this study should be limited to similarly-sized academic medical institutions in the same geographic region. Lastly, we were unable to further analyze differences in gender for those accessing reproductive healthcare due to a small sample size of non-female identifying trainees; therefore, future studies among multiple institutions may be able to further analyze the needs and barriers of this group.

Future studies should expand on this study and investigate the trends amongst trainees at institutions across the county. To generalize the study results to institutions across the country, trainees across the country would need to be surveyed, as prior studies have shown that there is variability in healthcare access between institutions [14]. In addition, action toward identifying and reducing the barriers to gynecologic care access identified in this study should be implemented, and their impact should be evaluated.

## Conclusions

Female physicians experience infertility at a much higher rate than the general population. Unique time constraints and privacy concerns may be preventing medical trainees' access to OBGYN care, which poses a risk to their future fertility. This study provides insights into the care-seeking practices of medical students and residents regarding OBGYN care. The findings suggest that medical trainees access OBGYN care at low rates. Many medical students and residents are not up to date on recommended OBGYN care such as STI testing and cervical cancer screening, showing that there is a need to increase access for this population. Age, trainee type, and race may also be factors influencing the lack of access to OBGYN care. Routine gynecologic care is imperative to address infertility and reproductive planning. Increased education, improved resources, and increased time for medical appointments may reduce the barriers to accessing OBGYN care that were seen in this study.

## Appendices

Appendix A 

The questionnaire presented to the participants in this study is featured in Table 4.

**Table 4:** Questions for the medical trainees AMC: Albany Medical College, OBGYN: Obstetrics and gynecology, STI: Sexually transmitted infection

Survey question	
Are you a medical student or resident at Albany Medical College? (Inclusion criteria)	
What year in medical school are you? (Medical Student)	What year are you? (Resident)
Do you have a uterus? (Inclusion criteria)	
What is your age?	
Are you on AMC's health insurance plan (MVP)?	
Are you enrolled on the AMC health insurance plan (CDPHP)?	
What is your race?	
What is your ethnicity?	
How do you currently describe your gender identity?	
Which of the following best represents your sexual orientation?	
Which of the following specialties are you most interested in pursuing (select up to three)? (Medical Student)	What department are you in? (Resident)
Have you EVER seen an OBGYN Provider?	
*If yes:* Have you seen an OBGYN provider in the last 12 months?	
*If no:* Have you seen an OBGYN provider in the last 3 years?	
Have you seen an OBGYN provider since you started your training at AMC?	
Did you switch to a new OBGYN provider when you started at AMC?	
To your best knowledge, have you had a Pap smear in the last 3 years? (<33 y/o)	
To your best knowledge, have you had a Pap smear in the last 5 years? (>33 y/o)	
Have you ever had sex of any kind (oral, vaginal, or anal)?	
*If yes:* Have you had sex in the last year?	
Do you have sex with someone who could get you pregnant?	
*If yes:* How often do you use a barrier when engaging in any type of sexual activity? (5-point scale: always to never)	
Have you ever been tested for sexually transmitted infections (STIs)?	
*If yes:* Have you received STI testing in the last year?	
*If yes:* Have you had sex with a new partner since you were last STI tested?	
Do you consider yourself to be at risk for getting an STI?	
*If no:* Why do you think you are at low risk for STIs?	
Since any previous partner(s), have you and your current partner been screened for STIs?	
Have you used any of the following in the last year (check all that apply)? (List of birth control methods)	
Was your birth control prescription or device prescribed by an AMC Provider?	
Have you ever ordered a prescription online without speaking face-to-face with a provider (telehealth or in person)?	
*If yes:* Have you ever ordered a contraception prescription or abortion pill online without speaking face-to-face with a provider (telehealth or in person)?	
In the last year, did you tend to have a regular menstrual cycle (once every 24-38 days, with menses lasting 2-7 days)?	
*If no: *Do you have a known condition or are you on a medication that affects your menstrual cycle?	
